# Allosteric modulation of G protein‐coupled receptors by amiloride and its derivatives. Perspectives for drug discovery?

**DOI:** 10.1002/med.21633

**Published:** 2019-09-08

**Authors:** Arnault Massink, Tasia Amelia, Alex Karamychev, Adriaan P. IJzerman

**Affiliations:** ^1^ Division of Drug Discovery and Safety Leiden Academic Centre for Drug Research Leiden The Netherlands

**Keywords:** allosteric modulation, amiloride, drug discovery, G protein‐coupled receptors, 5‐(*N,N*‐hexamethylene)amiloride

## Abstract

The function of G protein‐coupled receptors (GPCRs) can be modulated by compounds that bind to other sites than the endogenous orthosteric binding site, so‐called allosteric sites. Structure elucidation of a number of GPCRs has revealed the presence of a sodium ion bound in a conserved allosteric site. The small molecule amiloride and analogs thereof have been proposed to bind in this same sodium ion site. Hence, this review seeks to summarize and reflect on the current knowledge of allosteric effects by amiloride and its analogs on GPCRs. Amiloride is known to modulate adenosine, adrenergic, dopamine, chemokine, muscarinic, serotonin, gonadotropin‐releasing hormone, GABA_B_, and taste receptors. Amiloride analogs with lipophilic substituents tend to be more potent modulators than amiloride itself. Adenosine, α‐adrenergic and dopamine receptors are most strongly modulated by amiloride analogs. In addition, for a few GPCRs, more than one binding site for amiloride has been postulated. Interestingly, the nature of the allosteric effect of amiloride and derivatives varies considerably between GPCRs, with both negative and positive allosteric modulation occurring. Since the sodium ion binding site is strongly conserved among class A GPCRs it is to be expected that amiloride also binds to class A GPCRs not evaluated yet. Investigating this typical amiloride‐GPCR interaction further may yield general insight in the allosteric mechanisms of GPCR ligand binding and function, and possibly provide new opportunities for drug discovery.

**Abbreviations**
5‐HT: 5‐hydroxy‐tryptamine
8‐OH‐DPAT: 8‐hydroxy‐2‐(di‐n‐propylamino)tetralin
B_max_: maximum number of binding sites
k_off_: dissociation rate constant
A‐EIA‐AS: (*N*‐2‐aminoethyl‐*N*‐isopropyl)amiloride‐*N*‐(4‐azidosalicylamide)
AB‐MECA: N6‐(4‐aminobenzyl)‐N‐methylcarboxamidoadenosine
BLT1: leukotriene B4 receptor
CBDMB: 5‐(N‐4‐chlorobenzyl)‐2′,4′‐dimethylbenzamil
CCL2: C–C motif chemokine ligand 2
CCR2: C–C chemokine receptor type 2
Cryo‐EM: cryogenic electron microscopy
DCB: 3′,4′‐dichlorobenzamil
DMA: 5‐(N,N‐dimethyl)amiloride
DPCPX: dipropylcyclopentylxanthine
EC_50_: half‐maximal effective concentration
EIA: 5‐(N‐ethyl‐N‐isopropyl)amiloride
E_max_: maximum efficacy
EMPA: N‐ethyl‐2‐[(6‐methoxy‐pyridin‐3‐yl)‐(toluene‐2‐sulfonyl)‐amino]‐N‐pyridin‐3‐yl‐methyl‐acetamide
FD‐1: furan derivative‐1
GABA_B_: γ‐aminobutyric acid‐B
GnRH: gonadotropin‐releasing hormone
GPCRs: G protein‐coupled receptors
GTPγS: guanosine 5′‐O‐(γ‐thio)triphosphate
hA_2A_AR: human adenosine A_2A_ receptor
HMA: 5‐(*N*,*N*‐hexamethylene)amiloride
IC_50_: half‐maximal inhibitory concentration
K_i_: equilibrium inhibition constant
LTB_4_: leukotriene B_4_
MBA: 5‐(N‐methyl‐N‐butyl)amiloride
MGCMA: 5‐(N‐methyl‐N‐guanidinocarbonyl‐methyl)amiloride
MIBA: 5‐(N‐methyl‐N‐isobutyl)amiloride
NECA: 5′‐(N‐ethylcarboxamido)adenosine
NMR: nuclear magnetic resonance
OX_2_R: orexin‐2 receptor
PIA: (‐)‐N6‐(R‐phenylisopropyl)‐adenosine
SEM: standard error of the mean
T1R2: taste receptor type 2
T1R2‐HD: taste receptor type 2‐heptahelical domain
T1R3: taste receptor type 3
WT: wild‐type

## 1 INTRODUCTION

G protein‐coupled receptors (GPCRs) form a family of receptors with approximately 800 members that are responsible for many different physiological functions such as regulation of sleep, vision, blood pressure, central nervous system activity, taste, and olfaction.^1^ This is reflected by the fact that they are directly or indirectly targeted by 30% to 40% of therapeutic drugs currently in the market.^2^, ^3^ GPCRs are grouped according to their structural and genomic characteristics in five main groups: rhodopsin‐like (class A), secretin‐like (class B), glutamate‐like (class C), adhesion, and frizzled/taste2, with class A being the largest group.^4^, ^5^

The precise mechanisms of action of these receptors have been studied for a long time, but due to the complexity of their structures, they are not yet fully understood. Novel pharmacological concepts have been introduced that reflect this complexity. For the purpose of this review, the concept of allosteric modulation is particularly relevant, which has been excellently reviewed elsewhere.^6^, ^7^, ^8^ The recent increase in high‐resolution GPCR crystal and cryo‐EM structures also allows a better understanding of how GPCRs function.^9^, ^10^, ^11^ Cocrystallization with orthosteric ligands such as agonists and antagonists allows the study of the orthosteric binding sites, that is the sites for endogenous hormones and neurotransmitters. However, to study allosteric binding sites cocrystallization with allosteric modulators is desired, which is a challenge due to their often low affinities. Adding high concentrations of sodium ions is a common procedure in the crystallization of GPCRs to stabilize the protein, which makes it possible for these ions to bind to low‐affinity sites. However, sodium ions are relatively small and need a high resolution (<2 Å) to be visualized. In recent crystal structures of several GPCRs the resolution was sufficiently high to locate a sodium ion bound in a site which is highly conserved amongst class A GPCRs.^12^ Currently solved crystal structures with a sodium ion bound in this allosteric site are of the human adenosine A_2A_ receptor,^13^ the β_1_‐adrenergic receptor,^14^, ^15^ the human δ‐opioid receptor,^16^ and the human protease‐activated receptor 1.^17^ The common residues that interact with the sodium ion in these crystal structures, either directly or through water‐mediated hydrogen bond interactions, are Asp^2.50^, Ser^3.39^ Trp^6.48^, Asn^7.45^, and Asn^7.49^ (numbering according to Ballesteros‐Weinstein^18^). The negatively charged amino acid Asp^2.50^ makes a strong salt bridge with the positively charged sodium ion and is essential for its binding in this site, which confirmed previous “pre‐crystal structure” research.^19^ It is also the most conserved residue of the sodium ion site amongst GPCRs. The high conservation of the sodium ion pocket amongst class A GPCRs makes it probable that more structures with sodium ions bound in this site will emerge. There is little if any conservation present in the other GPCR classes, which makes it improbable that such a sodium ion binding site exists in these GPCRs.

**Figure 1:**
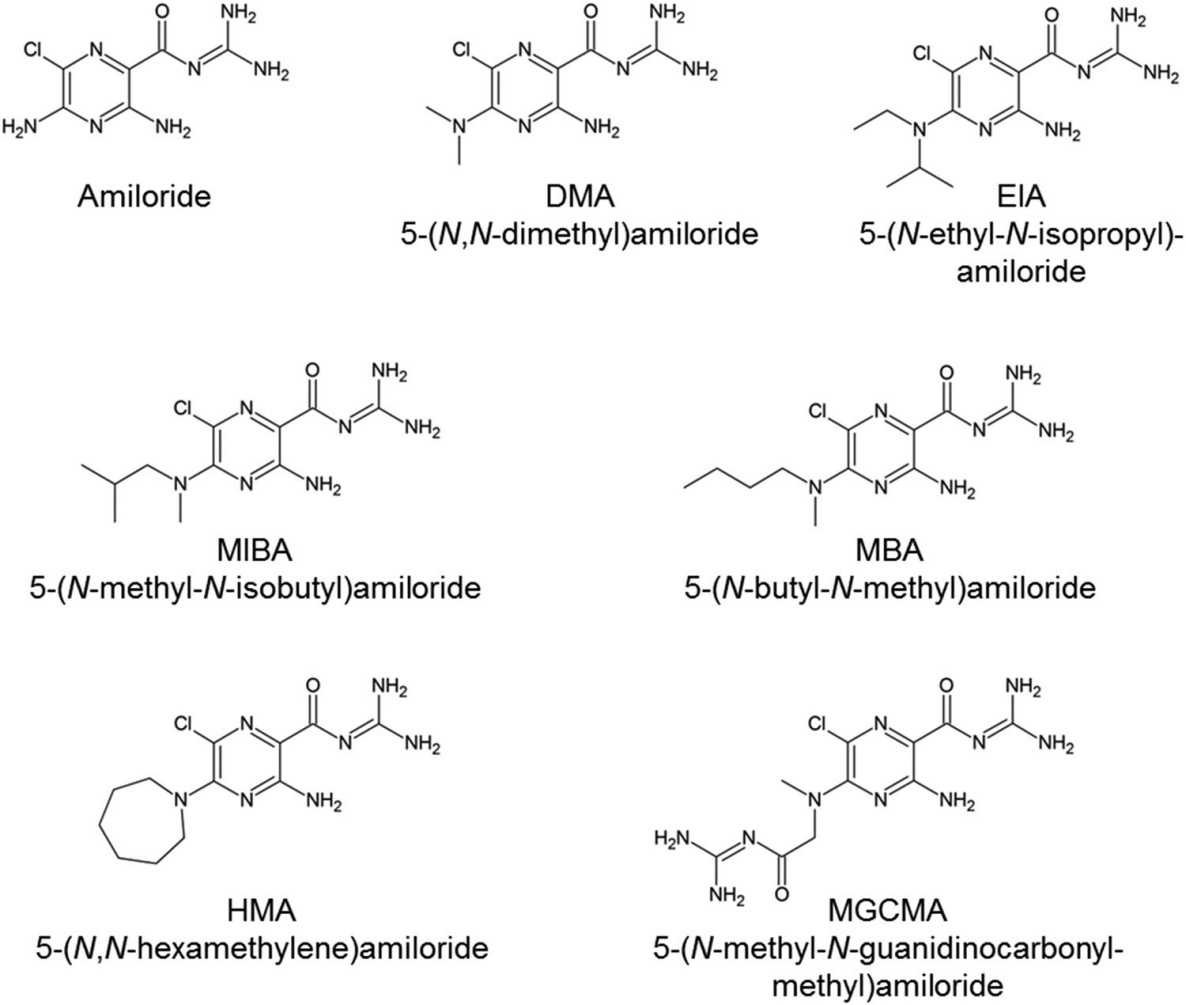
Chemical structures of amiloride and its 5′‐amino substituted analogs DMA, EIA, MIBA, MBA, HMA, and MGCMA. DMA, 5‐(*N*,*N*‐dimethyl)amiloride; EIA, 5‐(*N*‐ethyl‐*N*‐isopropyl)amiloride; HMA, 5‐(*N*,*N*‐hexamethylene)amiloride; MBA, 5‐(*N*‐methyl‐*N*‐butyl)amiloride; MGCMA, 5‐(*N*‐methyl‐*N*‐guanidinocarbonyl‐methyl)amiloride; MIBA, 5‐(*N*‐methyl‐N‐isobutyl)amiloride

**Figure 2:**
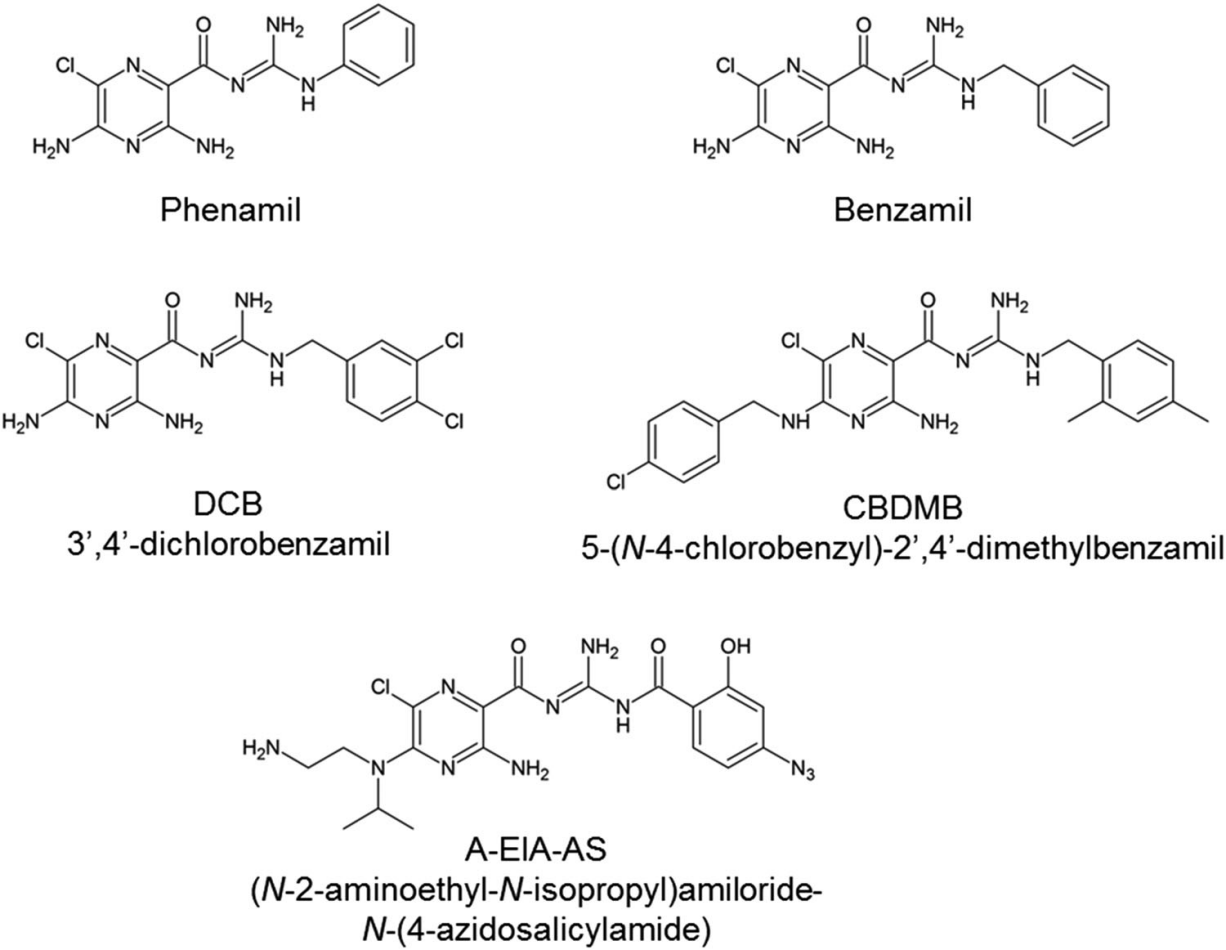
Chemical structures of 2‐guanidino substituted amiloride analogs phenamil, benzamil, DCB, CBDMB, and A‐EIA‐AS. A‐EIA‐AS, (*N*‐2‐aminoethyl‐*N*‐isopropyl)amiloride‐*N*‐(4‐azidosalicylamide); CBDMB, 5‐(*N*‐4‐chlorobenzyl)‐2',4'‐dimethylbenzamil; DCB, 3',4'‐dichlorobenzamil

Amiloride is primarily known as a potassium‐sparing diuretic drug, acting through the blockade of renal epithelial sodium channels.^20^ Amiloride and its analogs have also been found to bind to the sodium ion site of several GPCRs, modulating orthosteric ligand binding.^21^ The negatively charged carboxylate of sodium ion site residue Asp^2.50^ supposedly interacts with the positively charged guanidinium group present in all amilorides. The binding of amilorides into the sodium ion site of class A GPCRs renders these compounds potential pharmacological tools to probe molecular mechanisms of GPCR allosteric modulation. The chemical structures of amiloride and its analogs discussed in this review are depicted in Figures 1, 2. Effects of the amilorides are represented in Table 1 categorized per GPCR and orthosteric ligands used. Most of the receptors in Table 1 are discussed in the main text.

**Table 1:** Modulation of G protein‐coupled receptors by amiloride and amiloride analogs. The given values reflect a) inhibitory potency or affinity for ligand displacement in radioligand binding assays or inhibition of ligand‐induced receptor activation in functional assays, b) modulatory potency of their effect on radioligand dissociation, and c) fold change of dissociation rates of orthosteric ligands. References are in numbered superscript

Receptor	Orthosteric ligand antagonist; agonist	Amiloride (analog)	Inhibitory potency or affinity^a^ ‐ Displacement of orthosteric ligand by amiloride (analog) in IC_50_ or K_i_ ± *SEM* (µM)	Modulatory potency ‐ Concentration‐effect on dissociation of orthosteric ligand by amiloride (analog) in EC_50_ ± *SEM* (µM)	Effect on dissociation of orthosteric ligand by 100 µM^b^ amiloride (analog) in k_off_/k_off(control)_ > 1, Increase < 1, Decrease
Adenosine A_1_	[^3^H]DPCPX	Amiloride	2.0 ± 0.2^*B*24^	…	1.5^*Rd*26^
			197 ± 23^*R*26^		
		Benzamil	0.65 ± 0.04^*B*24^	…	…
		CBDMB	1.2 ± 0.1^*B*24^	…	…
		DCB	1.6 ± 0.1^*B*24^	…	…
		DMA	8 ± 2^*R*26^	…	1.9^*R*26^
		HMA	0.41 ± 0.03^*B*24^	…	1.7^*R*26^
			22 ± 4^*R*26^		
		MBA	0.070 ± 0.004^*B*24^	…	…
		MGCMA	22 ± 1^*B*24^	…	…
		MIBA	0.16 ± 0.01^*B*24^	…	…
			13 ± 1^*R*26^		
		Phenamil	1.5 ± 0.1^*B*24^	…	…
	*[* ^*3*^ *H]PIA*	Amiloride	2.4 ± 0.1^*B*24^	…	No effect^*Rd*26^
		Benzamil	0.85 ± 0.03^*B*24^	…	…
		CBDMB	4.0 ± 0.4^*B*24^	…	…
		DCB	2.7 ± 0.2^*B*24^	…	…
		DMA	…	…	No effect^*R*26^
		HMA	0.50 ± 0.03^*B*24^	…	No effect^*R*26^
		MBA	0.09 ± 0.01^*B*24^	…	…
		MGCMA	16 ± 1^*B*24^	…	
		MIBA	0.20 ± 0.01^*B*24^	…	…
		Phenamil	2.3 ± 0.1^*B*24^	…	…
Adenosine A_2A_	[^3^H]ZM‐241,385	Amiloride	9.7 ± 1.1^*R*25^	…	1.2^*Rd*25^
		Benzamil	2.2 ± 0.3^*R*25^	…	2.4^*Rd*25^
		HMA	3.3 ± 0.5^*R*25^	…	12^*Rd*25^
		MGCMA	89 ± 13^*R*25^	…	1.2^*Rd*25^
		MIBA	3.0 ± 0.2^*R*25^	…	5.7^*Rd*25^
		Phenamil	2.6 ± 0.4^*R*25^	…	1.9^*Rd*25^
	*[* ^*3*^ *H]CGS‐21,680*	Amiloride	…	…	No effect^*Rd*26^
		DMA	…	…	No effect^*R*26^
		HMA	…	…	No effect^*R*26^
Adenosine A_3_	[^3^H]PSB‐11	Amiloride	82 ± 7^*H*26^	…	No effect^*Hd*26^
		DMA	13 ± 2^*H*26^	…	1.3^*H*26^
		HMA	6 ± 1^*H*26^	…	2.3^*H*26^
		MIBA	8 ± 1^*H*26^	…	1.6^*H*26^
	*[* ^*125*^ *I]I‐AB‐MECA*	Amiloride	>100^*R*26^	…	No effect^*Hd*26^
		DMA	20 ± 3^*R*26^	…	*0.80* ^*H*26^
		HMA	7 ± 1^*R*26^	…	*0.53* ^*H*26^
		MIBA	7 ± 2^*R*26^	…	*0.59* ^*H*26^
α_1A_‐Adrenergic	[^3^H]Prazosin	Amiloride	11 ± 2^*H*37^	…	1.2^*H*37^
		Benzamil	0.8 ± 0.1^*H*37^	…	1.7^*H*37^
		DMA	0.82 ± 0.03 ^*H*37^	…	1.5^*H*37^
		EIA	2.7 ± 0.3 ^*H*37^	…	2.2^*H*37^
		HMA	1.1 ± 0.2^*H*37^	…	5.5^*H*37^
		MIBA	0.49 ± 0.07^*H*37^	…	2.4^*H*37^
α_2A_‐Adrenergic	[^3^H]Yohimbine	Amiloride	30 ± 2^*H*42^	…	2.0^*He*42^
		A‐EIA‐AS	…	40^*P*41^	>1^*P*41^
		Benzamil	3.5 ± 0.7^*H*42^	…	No effect^*He*42^
		DMA	3.6 ± 0.1^*H*42^	…	5.3^*He*42^
	[^3^H]Rauwolscine		…	…	6.3^*He*42^
	[^3^H]RX‐821,002		…	…	7.1^*He*42^
	[^3^H]Yohimbine	EIA	1.7 ± 0.2^*H*42^	50^*P*41^	>1^*P*41^
					155^*He*42^
		HMA	0.21 ± 0.00^*H*42^	…	138^*He*42^
	[^3^H]Rauwolscine		…	…	57^*He*42^
	[^3^H]Yohimbine	MIBA	0.56 ± 0.01^*H*42^	…	101^*He*42^
	*[* ^*3*^ *H]UK‐14,304*	Amiloride	25 ± 0.2^*H*43^	…	*0.67* ^*He*43^
		DMA	3.2 ± 0.2^*H*43^	…	*0.77* ^*He*43^
		HMA	0.18 ± 0.02^*H*43^	…	*0.37* ^*He*43^
α_2B_‐Adrenergic	[^3^H]Rauwolscine	CBDMB	…	…	*<1* ^*R*44^
		EIA	…	…	>^*R*44^
		MIBA	…	…	>1^*R*44^
β_1_‐Adrenergic	[^125^I]Iodocyano‐pindolol	Amiloride	83 ± 14^*R*36^	…	…
β_2_‐Adrenergic	[^125^I]Iodocyano‐pindolol	Amiloride	60^*R*36^	…	…
CCR_2_	[^3^H]INCB3344	Amiloride	No effect^*Hf*45^	…	…
		Benzamil	No effect^*Hf*45^	…	…
		HMA	79^*H*45^	…	1.25^*H*45^
		MCGMA	No effect^*Hf*45^	…	…
		MIBA	158^*H*45^	…	…
		Phenamil	No effect^*Hf*45^	…	…
	[^3^H]CCR2‐RA‐[R]^c^	Amiloride	No effect^*Hf*45^	…	…
		Benzamil	No effect^*Hf*45^	…	…
		HMA	79^*H*45^	…	1.36^*H*45^
		MCGMA	No effect^*Hf*45^	…	…
		MIBA	126^*H*45^	…	…
		Phenamil	No effect^*Hf*45^	…	…
	*[* ^*125*^ *I]CCL2*	HMA	…	…	9.7^*H*45^
Dopamine D_1_	[^3^H]SCH‐23,390	Amiloride	49 ± 1^*H*50^	>1000^*H*50^	…
		Benzamil	1.6 ± 0.5^*H*50^	74 ± 8^*H*50^	…
		MIBA	4.4 ± 0.2^*H*50^	13 ± 1^*H*50^	26^*He*50^
Dopamine D_2_	[^125^I]Epidepride	Amiloride	…	…	2.5^*Ri*51^
	[^3^H]Spiperone		390 ± 4^*H*50^	215 ± 35^*R*53^	1.5^*Ri*51^
			…	100 ± 10^*H*50^	2.7^*Rj*53^
		Benzamil	25 ± 2^*H*50^	46 ± 4^*R*53^	4.8^*Rk*53^
				29 ± 7^*H*50^	
		DMA	…	76 ± 8^*R*53^	8.4^*Rk*53^
		EIA	…	20 ± 5^*R*53^	18^*Rl*53^
		HMA	…	10 ± 2^*R*53^	16^*Rl*53^
		MIBA	6.6 ± 0.4^*H*50^	14 ± 1^*R*53^	14^*Rl*53^
				2.1 ± 0.2^*H*50^	88^*He*50^
	*Dopamine*	Amiloride	29^*Rm*54^	…	…
		DMA	1.4^*Rm*54^	…	…
		MIBA	0.9^*Rm*54^		…
			0.6 ± 0.2^*Hn*50^		
Dopamine D_3_	[^3^H]Spiperone	Amiloride	120 ± 7^*H*50^	43 ± 3 ^*H*50^	…
		Benzamil	16 ± 1^*H*50^	15 ± 2^*H*50^	…
		MIBA	1.7 ± 0.1^*H*50^	0.29 ± 0.14^*H*50^	18^*He*50^
	*Dopamine*	MIBA	1.8^*Ro*54^	…	…
Dopamine D_4_	[^3^H]Spiperone	Amiloride	280 ± 30^*H*50^	420 ± 4^*H*50^	…
		Benzamil	6.1 ± 0.4^*H*50^	28 ± 2^*H*50^	…
		MIBA	1.3 ± 0.2^*H*50^	22 ± 5^*H*50^	>1^*He*50^
GnRH	*[* ^*125*^ *I]Triptorelin*	Amiloride	>100^*H*63^	…	…
		Benzamil	>100^*H*63^	…	…
		DCB	30 ± 3 ^*H*63^	…	1.7^*H*63^
		MIBA	39 ± 7^*H*63^	…	2.1^*H*63^
		HMA	29 ± 3 ^*H*63^	49 ± 7^*H*63^	2.5^*H*63^
		MCGMA	>100^*H*63^	…	…
		Phenamil	>100^*H*63^	…	…
Histamine H_1_	[^3^H]Mepyramine	Amiloride	>10^*R*21^	…	…
		Benzamil	3.2 ± 0.2^*R*21^	…	…
		HMA	5.6 ± 1.2^*R*21^	…	…
Muscarinic M_1_	[^3^H]Pirenzepine	Amiloride	>10^*R*21^	…	…
		Benzamil	2.9 ± 0.7^*R*21^	…	…
		HMA	3.6 ± 1.0^*R*21^	…	…
Muscarinic M_2_	[^3^H]*N‐*methyl‐scopolamine	Amiloride	>10^*R*21^	…	…
		Benzamil	5.8 ± 1.1^*R*21^	…	…
		HMA	2.9 ± 0.5^*R*21^	…	…
Muscarinic M_3_	[^3^H]*N‐*methyl‐scopolamine	Amiloride	50^*R*67^	…	…
		Benzamil	2.8 ± 0.5^*R*21^	…	…
		HMA	4.7 ± 0.8^*R*21^	…	…
	*Acetylcholine*	Amiloride	478^*Rq*65^	…	…
δ‐Opioid	*[* ^*3*^ *H]DADLE*	Amiloride	>10^*R*21^	…	…
		Benzamil	>10^*R*21^	…	…
		HMA	1.0 ± 0.2^*R*21^	…	…
κ‐Opioid	*[* ^*3*^ *H]Ethyl‐ketazocine*	Amiloride	>10^*R*21^	…	…
		Benzamil	>10^*R*21^	…	…
		HMA	3.9 ± 0.6^*R*21^	…	…
µ‐Opioid	[^3^H]Naloxone	Amiloride	>10^*R*21^	…	…
		Benzamil	1.1 ± 0.4^*R*21^	…	…
		HMA	0.06 ± 0.02^*R*21^	…	…
Serotonin 5‐HT_1A_	*[* ^*3*^ *H]8‐OH‐DPAT*	Amiloride	>10^*R*21^	…	…
		Benzamil	1.9 ± 0.3^*R*21^	…	…
		HMA	>10^*R*21^	…	…
Serotonin 5‐HT_1B_	*[* ^*3*^ *H]5‐Carboxa‐midotryptamine*	Amiloride	20^*H*73^	…	…
	*Sumatriptan*		35^*Hs*73^	…	…
	*[* ^*3*^ *H]Serotonin*	Benzamil	>10^*R*21^	…	…
	*[* ^*3*^ *H]5‐Carboxa‐midotryptamine*	EIA	13^*H*73^	…	…
	*[* ^*3*^ *H]Serotonin*	HMA	>10^*R*21^	…	…
Serotonin 5‐HT_1C_	*[* ^*3*^ *H]Serotonin*	Amiloride	>10^*R*21^	…	…
		Benzamil	>10^*R*21^	…	…
		HMA	6.7 ± 1.2^*R*21^	…	…
Serotonin 5‐HT_1D_	*[* ^*3*^ *H]Serotonin*	Amiloride	>10^*R*21^	…	…
		Benzamil	>10^*R*21^	…	…
		HMA	>10^*R*21^	…	…
Serotonin 5‐HT_2_	*[* ^*3*^ *H]Serotonin*	Amiloride	>10^*R*21^	…	…
		Benzamil	1.4 ± 0.1^*R*21^	…	…
		HMA	0.40 ± 0.06^*R*21^	…	…

Abbreviations: *B*, bovine receptor; CBDMB, 5‐(N‐4‐chlorobenzyl)‐2',4'‐dimethylbenzamil; *c*, in presence of 100 µM amiloride (analogue) except when stated otherwise; *d*, in presence of 1 mM amiloride (analog); DCB, 3',4'‐dichlorobenzamil; DMA, 5‐(N,N‐dimethyl)amiloride; *e*, calculated for the amiloride (analog) occupied receptor; EIA, 5‐(N‐ethyl‐N‐isopropyl)amiloride; *f*, no displacement of orthosteric ligand by 100 µM amiloride (analog); *g*, [3H]CCR2‐RA‐[R] is an ‘intracellular antagonist’ as it binds intracellularly to the chemokine CCR2 receptor; HMA, 5‐(N,N‐hexamethylene)amiloride; *H*, human receptor; MGCMA, 5‐(N‐methyl‐N‐guanidinocarbonyl‐methyl)amiloride; *i*, in presence of 500 µM amiloride (analog); *j*, in presence of 3.16 mM amiloride (analog); *k*, in presence of 1 mM amiloride (analog); MIBA, 5‐(N‐methyl‐N‐isobutyl)amiloride; *q*, modulation by amiloride of acetylcholine‐induced contractions of rat tracheal smooth muscle, which expresses the muscarinic M3 receptor; *R*, rat receptor; *m*, inhibition by amiloride (analog) of dopamine‐stimulated increase in extracellular acidification rate in cells expressing the dopamine D2 receptor; *n*, inhibition by MIBA of dopamine‐stimulated [35S]GTPγS binding to dopamine D2 receptors; *s*, inhibition by amiloride of the sumatriptan‐induced reduction of cAMP formation stimulated by forskolin in cells expressing the Serotonin 5‐HT1B receptor; SEM, standard error of mean.

^a^ IC_50_ values determined with concentrations of orthosteric radioligands around their K_D_.

^b^ In presence of 100 µM amiloride (analog) except when stated otherwise.

^c^ [^3^H]CCR2‐RA‐[R] is an ‘intracellular antagonist’ as it binds intracellularly to the chemokine CCR2 receptor.

## 2 ADENOSINE RECEPTORS

Adenosine receptors have been studied extensively, and as a result, many orthosteric^22^ and allosteric^23^ ligands have been discovered. Amiloride interactions with adenosine receptors were discovered in the early days of adenosine receptor research.^24^ Since the effects of amiloride binding to adenosine receptors appeared to be closely tied to sodium ion interactions, it was necessary to investigate and exclude the involvement of Na^+^/H^+^ exchange proteins (one of the main targets of amiloride) in these interactions.^21^ In this study, Garritsen et al^21^ found inhibition of antagonist [^3^H]DPCPX and agonist [^3^H]PIA at the calf adenosine A_1_ receptor by amiloride, its 5′‐amino‐substituted analogs 5‐(*N*,*N*‐hexamethylene)amiloride (HMA), 5‐(*N*‐methyl‐*N*‐butyl)amiloride (MBA), 5‐(*N*‐methyl‐*N*‐guanidinocarbonyl‐methyl) amiloride (MCGMA), and 5‐(*N*‐methyl‐*N*‐isobutyl)amiloride (MIBA), and its 2‐guanidino substituted analogs benzamil, 5‐(*N*‐4‐chlorobenzyl)‐2′,4′‐dimethylbenzamil (CBDMB), 3′,4′‐dichlorobenzamil (DCB), and phenamil.

**Figure 3:**
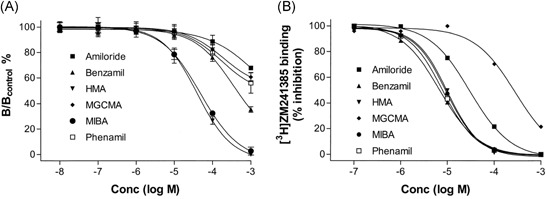
Concentration dependence of amiloride and its analogs for A, increase of [^3^H]ZM‐241,385 dissociation and B displacement of [^3^H]ZM‐241,385 after reaching binding equilibrium at adenosine A_2A_ receptors. In A, [^3^H]ZM‐241,385 binding was allowed to first reach equilibrium at the receptor before its dissociation was induced by addition of an excess of antagonist, in the absence and presence of increasing concentrations of amiloride (analog). The results are expressed as a ratio between the binding of [^3^H]ZM‐241,385 after 120 minutes in the presence (“B”) and in the absence (B_control_) of amiloride (analog). Reproduced with permission from Gao and IJzerman.^25^ HMA, 5‐(*N*,*N*‐hexamethylene)amiloride; MBA, 5‐(*N*‐methyl‐*N*‐butyl)amiloride; MGCMA, 5‐(*N*‐methyl‐*N*‐guanidinocarbonyl‐methyl)amiloride

Gao and IJzerman^25^ found that amiloride analogs benzamil, HMA, MCGMA, MIBA, and phenamil increased the dissociation rate of the antagonist [^3^H]ZM‐241,385 at the rat A_2A_ receptor, and that they were more potent than amiloride itself (Figure 3). However, the affinity (defined by radioligand displacement in equilibrium) and the allosteric potency (defined by the concentration‐dependent effect on the radioligand dissociation rate) did not correlate. This indicated a mixed competitive (ie, mutually exclusive displacement) and noncompetitive behavior of amilorides, in which amilorides and orthosteric ligands bind to the receptor at the same time, whereas amiloride influences the orthosteric ligand's dissociation rate. The amiloride analogs HMA and MIBA, with a lipophilic moiety on the 5′‐position, proved to be the most potent compounds in increasing the dissociation rate of the orthosteric ligand, whereas they had equal affinities to benzamil and phenamil in displacing it. In contrast to the effect of amilorides, sodium ions decreased the dissociation rate of [^3^H]ZM‐241,385. Still, sodium ions and HMA appeared to compete for the same allosteric site.

In a study by Gao et al^26^ it appeared that adenosine receptor agonizts and antagonists are differently affected by amilorides. Amilorides increased the dissociation rates of antagonists [^3^H]DPCPX at the rat adenosine A_1_ and [^3^H]PSB‐11 at the human A_3_ receptors, just as with [^3^H]ZM‐241,385 at the rat A_2A_ receptor. However, they did not affect the dissociation rates of agonizts [^3^H]*R*‐PIA from the rat A_1_ and [^3^H]CGS‐21,680 from the rat A_2A_ receptors. Amilorides decreased the dissociation rate of agonist [^125^I]‐AB‐MECA at the rat adenosine A_3_ receptor, revealing that amilorides can also act as positive allosteric modulators depending on the radiolabeled probe used.^26^ Furthermore the amilorides exhibited selectivity for the different adenosine receptor subtypes. Amiloride and 5‐(*N*,*N*‐dimethyl)amiloride (DMA) were more potent at the A_1_ receptor in accelerating antagonist dissociation, whereas HMA was the most potent at the A_2A_ receptor and to a lesser extent at the A_3_ receptor.

**Figure 4:**
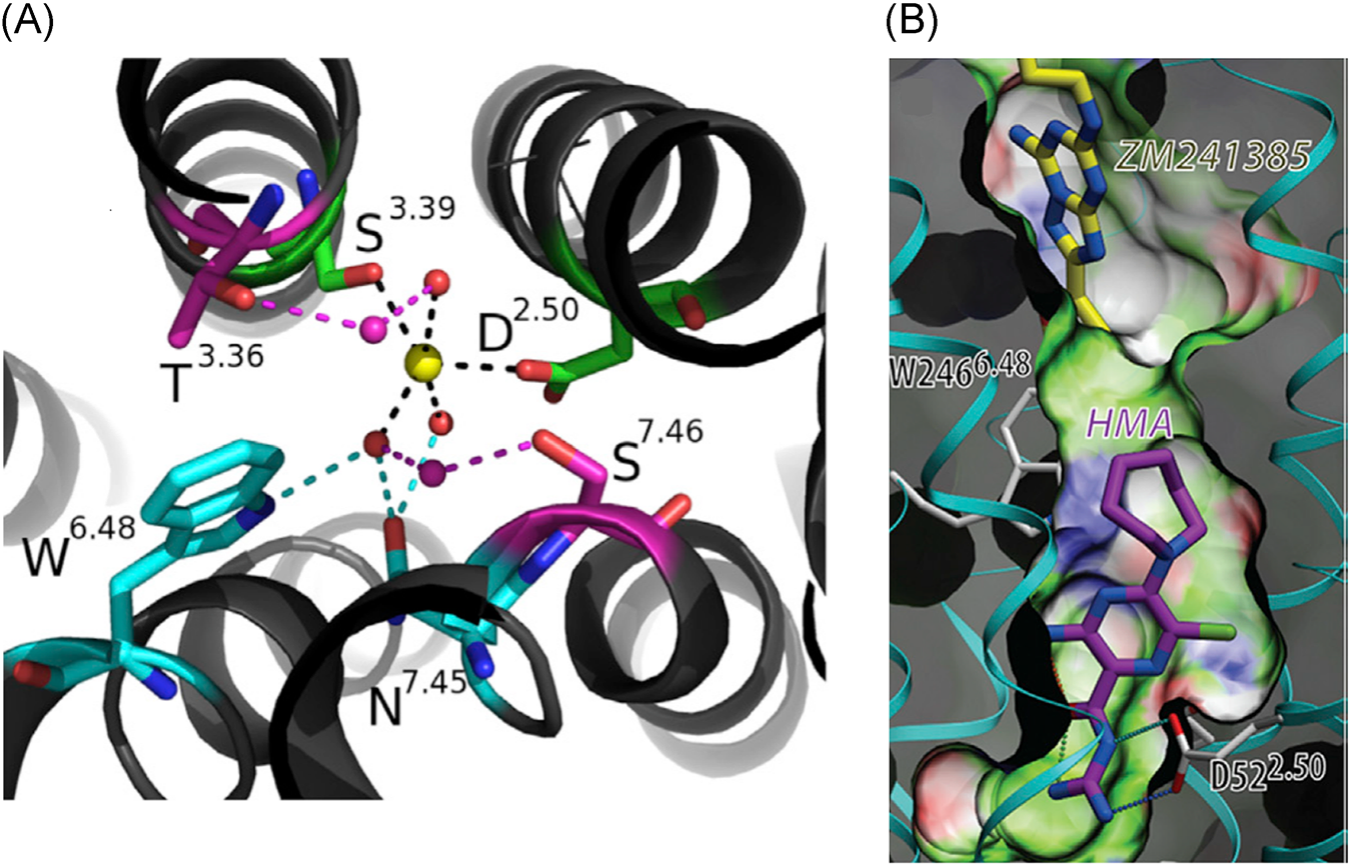
A, The Na^+^‐distorted octahedral coordination in the A_2A_AR crystal structure (PDB: 4EIY): the first shell is occupied by two conserved polar residues (green) and three water molecules (small spheres), which contact with the second shell of residues (cyan), or with a second layer of water molecules connecting with a third shell of residues (magenta). B, Docking of HMA in the sodium ion binding site. The guanidinium group of HMA has a salt bridge interaction with Asp52^2.50^ whereas the 5′‐azepane moiety of HMA clashes with Trp246^6.48^. ZM‐241,385 is the orthosteric antagonist. Reproduced with permission from Gutiérrez‐de Terán et al^27^ [Color figure can beviewed at wileyonlinelibrary.com]

**Figure 5:**
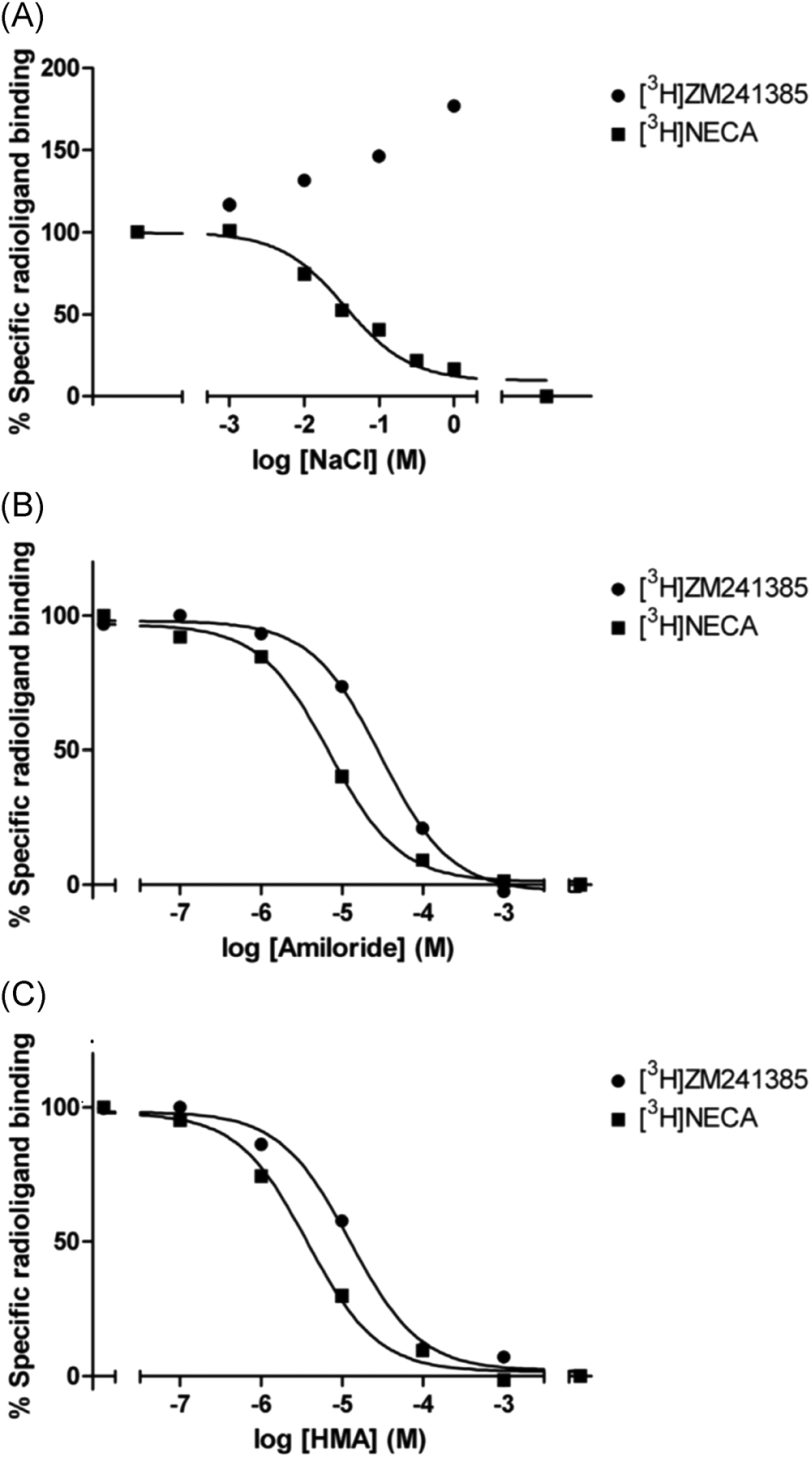
Equilibrium displacement of [^3^H]ZM‐241,385 (antagonist) and [^3^H]NECA (agonist) binding to A_2A_AR by allosteric modulators. A, NaCl, B, amiloride, and C, HMA. Reproduced with permission from Gutiérrez‐de Terán et al.^27^ HMA, 5‐(*N*,*N*‐hexamethylene)amiloride; NECA, 5'‐(*N*‐ethylcarboxamido)adenosine

Solving the crystal structure of the adenosine A_2A_ receptor at a resolution of 1.8 Å provided a sufficiently high resolution to detect a sodium ion bound in its allosteric binding site for the first time (Figure 4A).^13^ The amino acids interacting with the sodium ion in this site are highly conserved amongst other GPCRs which confirmed previous studies in which modulation by sodium ions was tied to the same amino acids for different GPCRs.^12^ The most conserved amino acid is a negatively charged aspartic acid (Asp52^2.50^) which interacts directly with the positively charged sodium ion by means of a salt bridge. In molecular dynamics simulations, Gutiérrez‐de‐Terán et al^27^ observed that the interaction of the sodium ion with Asp52^2.50^ is highly stable in the receptor's inactive conformation. The presence of the ion also avoids rotamer changes in two other highly conserved residues, Trp246^6.48^ and Asn280^7.45^. Interestingly, an active receptor conformation caused the site to contract to expel the sodium ion from this allosteric binding site. These calculations agree very well with radioligand binding studies on A_2A_AR (Figure 5A).^13^, ^27^, ^28^ Sodium ions induced an increase in [^3^H]ZM‐241,385 antagonist binding, but inhibited [^3^H]NECA agonist binding in a concentration dependent‐manner (Figure 5A),^27^ suggesting among others that the binding of agonist and sodium ions can be considered as “mutually exclusive”.^29^ Interestingly, the IC_50_ value of NaCl to inhibit agonist binding was approximately 50 mM, suggesting that under physiological conditions ([NaCl] = 140 mM) the receptor is predominantly in an inactive state.

The positively charged guanidinium moiety of amiloride and its analog HMA may also interact with Asp52^2.50^ in a manner similar to sodium ions, as inferred from docking studies (Figure 4B). Radioligand binding studies with antagonist [^3^H]ZM‐241 385 and agonist [^3^H]NECA demonstrated amiloride and more strongly so HMA to reduce radioligand binding, with greater potency on agonist binding for both (Figure 5B and 5C).^27^

In a subsequent study, Massink et al^30^ introduced amino acid mutations in the sodium ion binding site to assess the key residues in the interaction between amiloride/HMA and A_2A_AR.^30^ Mutation of the polar residues in the pocket was shown to either abrogate (D52A^2.50^ and N284A^7.49^) or reduce (S91A^3.39^, W246A,^6.48^ and N280A^7.45^) the negative allosteric effect of sodium ions on agonist binding. The D52A^2.50^ mutation also decreased the potency of amilorides with respect to ligand displacement, for example, an 18‐fold reduction in HMA's IC_50_ value for [^3^H]ZM‐241,385 binding. Conversely, a big potency gain was observed on the W246A^6.48^ mutant. HMA's IC_50_ value increased 25‐fold from 8.9 to 0.36 µM; a similar gain was observed for amiloride, from 63 to 2.6 µM. Apparently, this tryptophan residue, part of a so‐called activation micro‐switch,^31^ hinders amilorides to bind in hA_2A_R (and possibly other GPCRs). Indeed, at the adenosine A_3_ receptor, the mutation of Trp243^6.48^ into Ala increased the affinity of HMA as well.^32^

**Figure 6:**
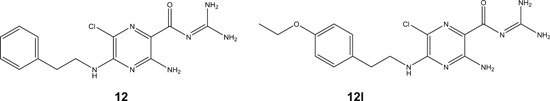
Amiloride derivatives **12** and **12l** 33

These findings fueled the ambition to design and synthesize novel amiloride/HMA derivatives. The 5′‐substitution of amiloride with phenylethyl (compound **12** in Massink et al^33^) yielded the largest decrease in antagonist [^3^H]ZM‐241,385 binding to both the wild‐type and W246A^6.48^ mutant receptors compared to other substituents and carbon chain elongations. Further derivatization of the phenylethyl moiety yielded 4‐ethoxyphenylethyl derivative **12l** (Figure 6), the most potent amiloride derivative of the series. This compound displaced [^3^H]ZM‐241,385 binding from the wild‐type A_2A_AR with an IC_50_ value of 3.4 µM, which was lower than HMA (5.1 µM). Derivative **12l** also showed an increased potency compared to that of HMA for the W246A^6.48^ mutant receptor, 19‐fold compared to WT for HMA in this study and 76‐fold for **12l**.^33^

The conformational flexibility of the adenosine A_2A_ receptor was examined further in a ^19^F NMR study, providing evidence for the occurrence of four different states of activation. Interestingly, both HMA and a partial agonist favored the population of an active state (S_3_), still different from the S_3′_ active state induced by full agonists.^34^ In a later study by the same team, the effects of NaCl were analyzed, leading to the conclusion that sodium ions reinforce an inactive ensemble of states (S_1‐2_), as well as the partial‐agonist, stabilized state (S_3_). HMA competed with the sodium ions, reflected in its effects on both line broadening and chemical shift perturbations in the ^23^Na NMR binding isotherm.^35^

In conclusion, the effects of amiloride and derivatives have been most extensively studied on adenosine A_2A_ receptors, through a number of orthogonal approaches. They all hint in the same direction, that is the amilorides compete with sodium ions at the allosteric sodium ion binding site in which Asp^2.50^ is the central amino acid. The evidence for other GPCRs is less exhaustive but suggests similar conclusions, which will be discussed below.

## 3 ADRENERGIC RECEPTORS

One of the first indications that amiloride inhibited the binding of orthosteric ligands at α‐ and β‐adrenergic receptors were found in 1987 by Howard et al,^36^ which was followed by many studies with amiloride and its analogs at a number of adrenergic receptor subtypes. At the human α_1A_‐adrenergic receptor amiloride and its analogs benzamil, DMA, 5‐(*N*‐ethyl‐*N*‐isopropyl)‐amiloride (EIA), MIBA, and HMA increased the dissociation rate of antagonist [^3^H]prazosin, and the analogs with bulky lipophilic 5′‐moieties were more potent in doing so.^37^, ^38^ Amiloride itself was characterized as an allosteric modulator acting at one allosteric site, but all the amiloride analogs appeared to bind to two different allosteric sites. The authors speculated that these allosteric sites could be present on one receptor or on a receptor dimer, but could not further confirm this.^37^ The allosteric interaction by amilorides was seemingly in contradiction with previous results at rat and mouse α_1_‐adrenergic receptors in which amiloride only showed a competitive interaction with antagonist [^3^H]prazosin binding but did not influence its dissociation rate.^36^

α_2_‐Adrenergic receptors are allosterically modulated by amilorides as well. At rat, human, bovine, and porcine α_2A_‐adrenergic receptors amiloride increased the dissociation rate of the antagonists [^3^H]rauwolscine^36^, ^39^ and [^3^H]yohimbine.^40^ Amiloride analogs also increased antagonist dissociation from the α_2A_‐adrenergic receptor, which was found for (*N*‐2‐aminoethyl‐*N*‐isopropyl)amiloride‐*N*‐(4‐azidosalicylamide; A‐EIA‐AS) at the porcine receptor,^41^ and DMA, EIA, MIBA, and HMA at the human receptor, in relation to [^3^H]yohimbine, [^3^H]rauwolscine, and [^3^H]RX‐821,002 dissociation.^42^ It is noteworthy that A‐EIA‐AS has no affinity for the Na^+^/H^+^ exchange protein, making it a GPCR selective amiloride. EIA, HMA, and MIBA were exceptionally strong negative allosteric modulators of antagonist binding, being 50‐ to 80‐fold more potent than amiloride in increasing the dissociation rate of [^3^H]yohimbine, showing that bulky lipophilic moieties at the 5′‐position of amiloride increase the allosteric potency at the α_2A_‐adrenergic receptor considerably. The apparent affinities of these amilorides were not correlating at all with their derived allosteric potencies in this study, cautioning to not confuse these two different pharmacological properties with each other.

In contrast to their effect on antagonists, amiloride, DMA, and HMA decreased the dissociation rate of agonist [^3^H]UK‐14 304 at the human α_2A_‐adrenergic receptor, with HMA having the largest effect.^43^ The dissociation‐slowing effect on agonist binding (2.7‐fold slower dissociation by HMA vs control) was considerably smaller though than the dissociation‐accelerating effect on antagonist binding (140‐fold faster dissociation by HMA). Although they slowed agonist dissociation, amilorides acted as negative allosteric modulators of α_2A_ receptor agonist activation, because amiloride, DMA, and HMA decreased the potency of norepinephrine and UK‐41,304 in [^35^S]GTPγS binding experiments. This paradoxical behavior was in line with previous findings that amilorides displace the orthosteric ligand competitively from the α_2A_ receptor in addition to their allosteric effects.^42^ Moreover, the addition of sodium ions increased the affinity of amiloride in doing so.^36^ This led to the conclusion that at α_2A_‐adrenergic receptors amilorides bind to two different sites, namely the orthosteric site and an allosteric sodium ion site. Howard et al^36^ hypothesized that amiloride binding in the orthosteric site was enhanced by binding of a sodium ion in the allosteric site, whereas amiloride binding in the allosteric site increased the dissociation rate of other orthosteric ligands. In a later study by Leppik et al,^42^ observed variations in the affinity of several amiloride analogs for the antagonist‐occupied and unoccupied receptor led to two different hypotheses. Either the amilorides bind to both the allosteric and orthosteric sites, or binding of an antagonist to the orthosteric site modified the conformation of the allosteric binding site in such a way that amiloride's affinity decreased.^42^

At the α_2B_ subtype, however, amilorides both increased and decreased the dissociation rate of antagonists. The 5′‐substituted amilorides EIA and MIBA increased the dissociation rate of [^3^H]rauwolscine binding, whereas the guanidino‐substituted amiloride CBDMB decreased it.^44^

The interaction of amiloride with β‐adrenergic receptors has only been studied by Howard et al in 1987. At both the β_1_‐ and β_2_‐adrenergic receptors amiloride displaced the antagonist [^125^I]iodocyanopindolol competitively, because their binding was mutually exclusive.^36^ Addition of sodium ions did not compete with amiloride binding, and it was concluded that amiloride did bind to the orthosteric site rather than to an allosteric sodium ion site. Despite the lack of modulation of β‐adrenergic receptors by sodium ions and amiloride, a sodium ion site was found in the crystal structure of the β_1_‐adrenergic receptor.^14^ The amino acids forming the sodium ion sites of the β_1_‐adrenergic and the adenosine A_2A_ receptor are the most similar of the solved GPCR crystal structures with such a site.^12^ That makes the difference in modulation by sodium ions and amilorides between these receptors remarkable and it is probably due to differences in the overall architecture of the two receptors.

## 4 CHEMOKINE RECEPTORS

Amiloride interactions with the chemokine receptor family have only been studied by Zweemer et al^45^ on the chemokine CCR2 receptor. The sodium ion site was the third binding site found on this receptor, next to the more extracellularly located orthosteric and an intracellular allosteric site.^46^, ^47^, ^48^, ^49^ Amiloride analogs MIBA and HMA inhibited binding of the antagonist [^3^H]INCB3344 binding to the orthosteric site and antagonist [^3^H]CCR2‐RA‐[R] binding to the intracellular site.^45^ Moreover, HMA inhibited binding of the orthosteric agonist [^125^I]CCL2. Amiloride, benzamil, MCGMA, and phenamil did however not displace any of these radioligands.

The increased dissociation rates of the orthosteric antagonist [^3^H]INCB3344, the intracellular antagonist [^3^H]CCR2‐RA‐[R], and the orthosteric agonist [^125^I]CCL2 induced by HMA indicate a noncompetitive allosteric interaction. Remarkably, the dissociation rate of the agonist [^125^I]CCL2 increased more (9.7‐fold) than of the antagonists (1.25‐ and 1.36‐fold) in the presence of HMA. Saturation binding assays revealed that HMA had a mixed competitive/noncompetitive interaction with the orthosteric antagonist [^3^H]INCB3344, because the radioligand's B_max_ value decreased and K_D_ value increased. HMA had a purely noncompetitive interaction with the intracellular antagonist [^3^H]CCR2‐RA‐[R], causing a decrease in this radioligand's B_max_ value only.

The allosteric effect of HMA was diminished by mutation of sodium ion site residues Asp88^2.50^ and His297^7.45^ into Ala. Mutation of Trp256^6.48^ even completely abolished HMA's allosteric effect, which is in contrast to the observed increase of HMA's affinity by the same mutation in adenosine receptors as discussed above.^32^ Amino acid His297^7.45^ is different from most class A GPCRs which usually harbor an Asn at the same position, but is conserved amongst chemokine receptors. The binding of HMA in CCR2s sodium ion binding site indicates that amiloride binding allows for a certain variation in the amino acids that constitute this binding cavity.

## 5 DOPAMINE RECEPTORS

The general trend amongst the dopamine receptor subtypes is an increase of the dissociation rate of orthosteric ligands by amiloride and its analogs, as found in a comprehensive study of the effect of amiloride, benzamil, and MIBA.^50^ MIBA had the largest effect on the dissociation rates of the antagonists [^3^H]SCH‐23,390 at the human D_1_ dopamine receptor and [^3^H]spiperone at the human D_2(short)_, D_2(long)_, D_3_, and D_4_ dopamine receptors. As with other GPCRs, the analogs with lipophilic moieties at the 5′‐position were more potent than amiloride itself. At the D_1_, D_2(short)_, D_2(long)_, and D_3_ dopamine receptors the amilorides displaced the orthosteric antagonist [^3^H]spiperone in both a noncompetitive and competitive manner. This may indicate binding of the amilorides to both the orthosteric and allosteric sites. The authors suggested a positive homotropic cooperativity due to a high Hill coefficient of the effect curves, i.e. amilorides binding at the allosteric site enhance the binding of amilorides to the orthosteric site.

**Figure 7:**
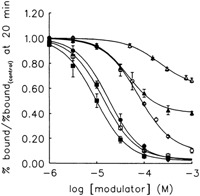
Concentration dependent dissociation modulation by amiloride and its analogs of [^3^H]spiperone binding at the dopamine D_2_ receptor after 20 minutes. (Δ‐amiloride, ▲‐benzamil, ○‐DMA, ●‐EIA, □‐MIBA, ■‐HMA). Amiloride modulates dissociation the least, whereas HMA and MIBA are the most effective modulators of dissociation. Reproduced with permission from Hoare and Strange.^53^ DMA, 5‐(*N*,*N*‐dimethyl)amiloride; EIA, 5‐(*N*‐ethyl‐*N*‐isopropyl)amiloride; HMA, 5‐(*N*,*N*‐hexamethylene)amiloride; MIBA, 5‐(*N*‐methyl‐*N*‐isobutyl)amiloride

The results at the D_2_ receptor complemented results from other studies, in which similar dissociation rate‐increasing effects and mixed competitive/noncompetitive behavior were found. Amiloride competed with and increased the dissociation rate of antagonists [^3^H]spiperone and [^125^I]epidepride binding.^51^ Amiloride, DMA, benzamil, EIA, MIBA, and HMA did so as well to the antagonist [^3^H]spiperone at both the rat^52^ and human^53^ D_2_ dopamine receptors, and of these amilorides HMA was the most potent amiloride (Figure 7). Agonists were modulated similarly as antagonists by amilorides at the rat D_2_ and D_3_ dopamine receptors, because amiloride, DMA, and MIBA decreased the potency of the agonist dopamine in inducing receptor activation in functional assays.^50^, ^54^ At the D_4_ receptor the allosteric effect of amiloride and its analogs was too small to be measured accurately, but an increase in antagonist [^3^H]spiperone dissociation rate was still detected. As amilorides still inhibited binding of the orthosteric ligand the displacement was more competitive in nature.^50^

The amino acids forming the sodium ion site in the dopamine receptors are conserved as well. Computational and mutagenesis studies at the D_2_ receptor have confirmed the importance of Asp80^2.50^, Ser121^3.39^, Asn419^7.45^, and Asn423^7.49^ for the allosteric effects by sodium ions.^55^, ^56^, ^57^ At the D_4_ dopamine receptor mutation of Asp80^2.50^ into Asn decreased MIBA affinity,^58^ indicating that amilorides bind in the sodium ion binding site as well. It may be assumed that amilorides also bind in the sodium ion binding site of the other dopamine receptors, but this has not been confirmed yet.

## 6 GONADOTROPIN‐RELEASING HORMONE RECEPTOR

The gonadotropin‐releasing hormone (GnRH) receptor, also known as luteinizing hormone‐releasing hormone receptor, is targeted by various drugs in the market for the treatment of sex‐hormone‐dependent diseases such as breast or prostate cancer.^59^, ^60^ These drugs are mostly peptidic agonists and antagonists that need to be administered by subcutaneous or intramuscular injections. The development of small‐molecule ligands that may replace these peptidic ligands is therefore desirable.^61^ Earlier results had indicated allosteric modulation of GnRH‐stimulated luteinizing hormone release by sodium ions and amilorides.^62^ In that light, the allosteric effects of amilorides on the GnRH receptor were investigated by Heitman et al^63^ Amiloride, benzamil, MCGMA, and phenamil had a negligible effect on the displacement of the peptide agonist [^125^I]triptorelin from the GnRH receptor. However, DCB, MIBA, and HMA increased the dissociation rate of [^125^I]triptorelin, with HMA having the strongest effect. In a luciferase assay, HMA acted as a purely insurmountable noncompetitive allosteric modulator as it only decreased the efficacy (E_max_) of GnRH receptor activation by triptorelin and the endogenous ligand GnRH. Furthermore, it was demonstrated that the GnRH receptor harbors a second allosteric site other than the amiloride binding site, because HMA did not compete with FD‐1, another allosteric modulator of the GnRH receptor with a distinct chemical structure.

## 7 MUSCARINIC RECEPTORS

Amiloride effects have been observed on muscarinic receptors in rat tissue preparations. Benzamil and HMA inhibited [^3^H]pirenzepine binding at the muscarinic M_1_ and [^3^H]*N*‐methylscopolamine binding at the muscarinic M_2_ and M_3_ receptors.^21^ In rat trachea amiloride inhibited muscarinic M_3_ receptor‐mediated smooth muscle contraction^64^ by the endogenous agonist acetylcholine, by an insurmountable noncompetitive interaction as its efficacy (E_max_) was reduced.^65^ In rat parotic acini, which express the muscarinic M_3_ receptor,^66^ amiloride inhibited binding of the muscarinic receptor antagonist [^3^H]N‐methylscopolamine in a competitive manner.^67^ In the recent, relatively low‐resolution crystal structures of the muscarinic M_2_ and M_3_ receptors sodium ion binding was not detected,^68^, ^69^, ^70^ but the amino acids making up the sodium ion site are perfectly conserved when compared to adenosine and adrenergic receptors,^12^ making amiloride binding to this site likely. In a recent molecular dynamics study sodium ion binding to (deprotonated), Asp^2.50^ in the muscarinic M_3_ receptor was suggested, keeping the receptor in an inactive state.^71^ Along a similar vein, the egress pathway of a sodium ion from Asp^2.50^ in the muscarinic M_2_ receptor into the cytosol was also simulated in molecular dynamics calculations.^72^

## 8 SEROTONIN RECEPTORS

Amiloride and analogs have been found to inhibit orthosteric ligand binding to serotonin receptors. Benzamil inhibited agonist [^3^H]8‐OH‐DPAT binding at the rat 5‐HT_1A_ receptor.^21^ Amiloride and EIA inhibited agonist [^3^H]5‐carboxamidotryptamine binding at the human 5‐HT_1B_ receptor.^73^ In functional assays at the same receptor, amiloride inhibited receptor activation by agonist sumatriptan in a competitive manner, whereas EIA displayed partial agonistic activity as it inhibited forskolin‐stimulated cAMP formation, albeit with a 15‐fold higher EC_50_ value (200 µM) compared to its K_i_ in inhibiting [^3^H]5‐carboxamidotryptamine binding (13 µM).^73^ Endogenous agonist [^3^H]serotonin binding was inhibited by HMA at the rat 5‐HT_1C_ receptor and by benzamil and HMA at the rat 5‐HT_2_ receptor.^21^ Crystal structures of the agonist bound 5‐HT_1B_ receptor^74^ and the 5‐HT_2B_ receptor,^75^ again at relatively low resolution, did not reveal a bound sodium ion, but the well‐conserved amino acids of the sodium ion site compared to the other class A GPCRs^12^ makes the binding of amiloride in the same location likely.

## 9 OREXIN RECEPTORS

Suno et al^76^ determined the crystal structure of the human orexin 2 (OX_2_) receptor in complex with the subtype‐selective antagonist *N*‐ethyl‐2‐[(6‐methoxy‐pyridin‐3‐yl)‐(toluene‐2‐sulfonyl)‐amino]‐*N*‐pyridin‐3‐yl‐methyl‐acetamide (EMPA) at 1.96 Å resolution.^76^ This high‐resolution structure enabled the authors to inspect the putative sodium ion binding site around Asp100^2.50^, better than in an earlier crystal structure of this receptor.^77^ Interestingly, and somewhat at odds with this review, the authors identified two water molecules rather than a sodium ion in the vicinity of this aspartic acid residue. Triggered by this absence they performed additional radioligand binding studies in which no effects were observed from the addition of sodium ions or amiloride derivatives, whereas such effects were found in a control experiment the authors performed on the hA_2A_R.

The receptors discussed above all belong to the class A family of GPCRs. Finally, we should like to discuss the evidence, admittedly limited and inconclusive, of amiloride interaction with two class C receptors.

## 10 GABA_B_ RECEPTORS

The GABA_B_ receptor is activated by γ‐aminobutyric acid (GABA) and it's derivative, baclofen (β‐4‐chlorophenyl‐GABA). This receptor is coupled to potassium and calcium channels through G_i_/G_o_ proteins.^78^ Ong and Kerr explored the interaction of amiloride and its analogs with baclofen‐induced depression of spontaneous discharges in rat isolated neocortical slices in Mg^2+^‐free medium. The effect of baclofen (10 µM) was blocked by amiloride (200 µM), which increased the frequency of discharges and slightly reduced their amplitude when applied alone. These effects persisted upon wash‐out and baclofen remained ineffective on the discharges until 30 to 60 minutes after a switch to amiloride‐free medium. Analogs of amiloride, DMA and MIBA, showed a similar mode of action, whereas they were at least twice as potent than amiloride in preventing the effect of baclofen on neocortical spontaneous discharges. DMA alone increased the discharge frequency and slightly reduced the amplitude in a concentration of 100 µM. Analogs lacking the guanidine moiety were ineffective. The authors explicitly stated, however, that an indirect effect of the amilorides via functional antagonism of coactivated adenosine A_1_ receptors cannot be ruled out.^79^

## 11 T1R2/T1R3 RECEPTORS

The heterodimeric T1R2 and T1R3 taste receptor acts as a sweet taste sensor with multiple binding sites for sweeteners.^80^ Amiloride (3 mM) were found to significantly reduce the responses to sweeteners such as sugar, artificial sweeteners, and sweet protein. Moreover, response inhibition of 1 mM aspartame by amiloride was observed in a concentration‐dependent manner with an IC_50_ value of 0.87 ± 0.20 mM. A study of the specificity towards the response mediated by the human sweet taste receptors showed that the suppression of receptor activity by amiloride is specific for hT1R2/hT1R3. Inhibitory effects of lactisole, a known hT1R2/hT1R3 inhibitor, and amiloride on the cellular response to aspartame were examined in cells expressing hT1R3 mutants (hT1R2/hT1R3‐A733V and hT1R2/hT1R3‐F778A). Lactisole was less active on the mutants, whereas amiloride did not show such a differential effect. These results suggest that the binding site of amiloride is distinct from that of lactisole.^81^ Amiloride inhibited the response of perillartine as a sweet activator on hT1R2/T1R3, T1R2, and T1R2‐heptahelical domain (HD). Molecular modeling suggested that perillartine and amiloride occupy the same binding pocket on the extracellular side of the hT1R2‐HD.^82^

## 12 FUTURE DIRECTIONS FOR DRUG DISCOVERY

It is increasingly realized that GPCRs have multiple binding sites that may influence each other in allosteric ways. The surge in crystal structures over the last decade has taught that ligands, including marketed drugs and clinical candidates, may have very different binding sites indeed. From this review, it has become obvious that the sodium ion binding site is yet another receptor domain to tune the ligand response, and that amiloride and its derivatives are prototypic small molecules that intervene with that site.

Does this offer options for future drug discovery? One might argue that the generic nature of the site and the evolutionary conservation of the amino acids aligning it are a drawback rather than an opportunity. In that view amilorides are another class of chemical probes that serve to unveil the complexities of GPCR functioning. A recent development, however, may prove this hypothesis wrong.

**Figure 8:**
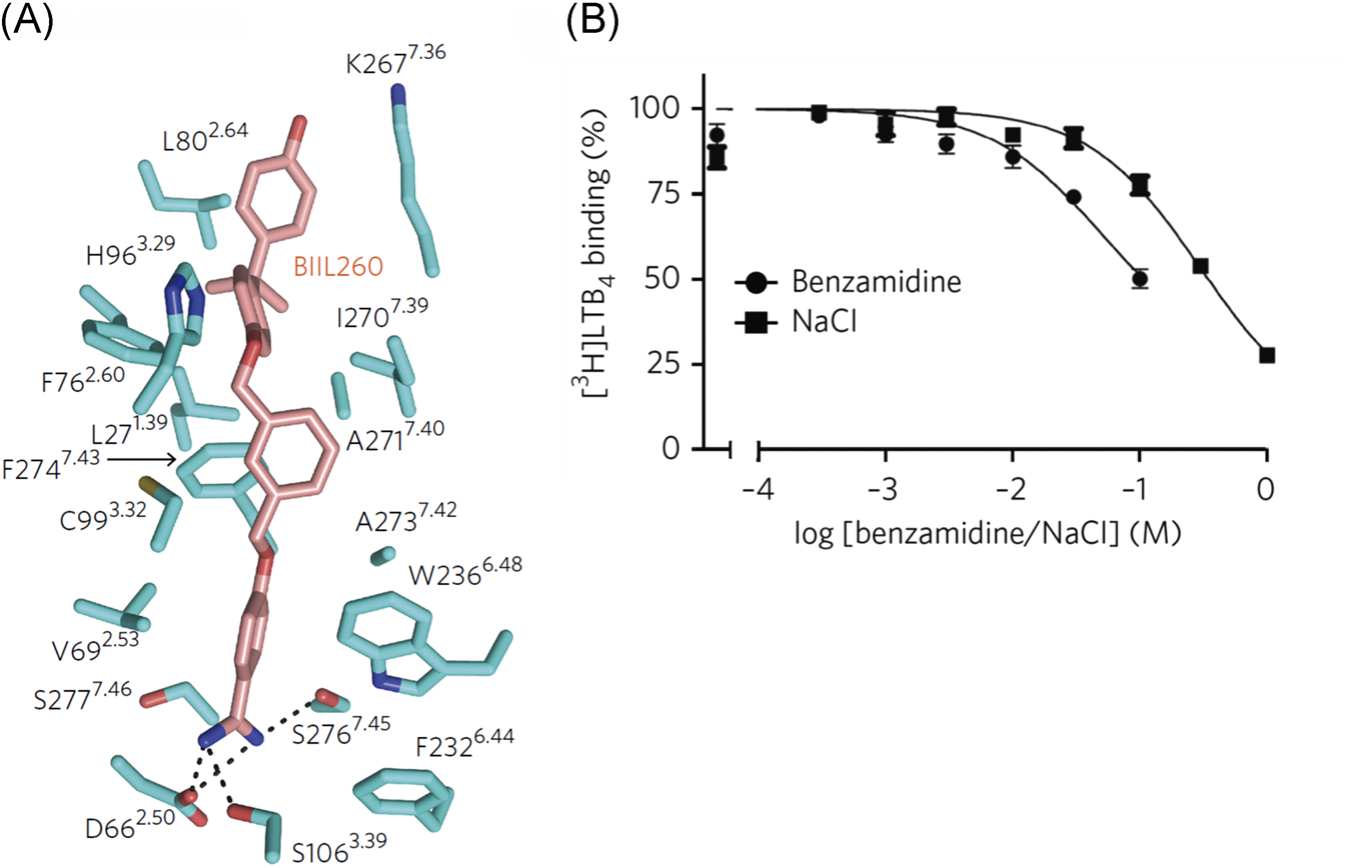
A, Structure of BIIL260 binding site in BLT1 receptor (PDB: 5X33); B, competition binding assay of benzamidine and NaCl to 0.5 nM [^3^H]LTB_4_. Reproduced with permission from Hori et al (2018).^83^ [Color figure can beviewed at wileyonlinelibrary.com]

The crystal structure of the leukotriene B_4_ (LTB_4_) receptor BLT1 in complex with antagonist/inverse agonist BIIL260 has recently been reported.^83^ Chemically, BIIL260 has four phenyl rings, three of which are bound in the orthosteric binding site near the extracellular domain. The fourth (a protonated benzamidine moiety) is penetrating deeper into the transmembrane domain and interacts with Asp66^2.50^, with which it forms a salt bridge. Hydrogen bonds are present with the hydroxyl groups of Ser106^3.39^ and Ser276^7.45^ (Figure 8A). Mutation of Asp^2.50^ or Ser^7.45^ to alanine markedly reduced the affinity of BIIL260 for the receptor providing also pharmacological evidence for the BIIL260's binding to the sodium ion binding site. Furthermore, benzamidine itself, as well as NaCl, served as negative allosteric modulators of radiolabeled agonist ([^3^H]LTB_4_) binding (Figure 8B), suggesting their capability of forcing the receptor in an inactive state.^83^ The chemical resemblance of amiloride's guanidine moiety and benzamidine might be a good starting point to further study the effects of amiloride and its analogs on the BLT1 receptor.

## 13 CONCLUDING REMARKS

This review summarizes the current knowledge of the allosteric effects of amiloride and its analogs on GPCRs. Allosteric effects of amilorides have been found on class A GPCRs (adenosine receptors, α‐adrenergic receptors, the CCR2 chemokine receptor, dopaminergic receptors, the gonadotropin‐releasing hormone receptor, the histamine H_1_ receptor, muscarinic receptors, opioid receptors, and serotonin receptors), and, less convincingly, on class C receptors (GABA_B_ and T1R2/3 receptors).

Amiloride and its analogs seem to follow a few general “rules” in their activity on these receptors. The propensity of amilorides to bind to the well‐conserved sodium ion site amongst GPCRs may explain these common behaviors. For most receptors, amiloride analogs with bulky lipophilic moieties on the 5′‐position have greater affinity and potency than the unsubstituted parent compound. This has not been explained fully, but it is clear that in most GPCRs there is a hydrophobic pocket above the sodium ion site that can accommodate these lipophilic moieties. Most receptors allow substitution on the guanidinium group as well, with a good affinity in displacing orthosteric ligands, but with less or no (allosteric) effect on the dissociation of orthosteric ligands.

Another general “rule” is the importance of Asp^2.50^ for amiloride binding, just as for sodium ions. In the docking studies performed, the binding mode of amiloride and HMA was predicted in the sodium ion site of the adenosine A_2A_ receptor crystal structure and a CCR2 chemokine receptor homology model. The positively charged guanidinium group has a strong salt bridge interaction with Asp^2.50^, underlining the great importance of this residue for amiloride binding as found before in mutagenesis studies. Trp^6.48^ interacts with amilorides as well, in some cases hampering and in other cases accommodating amiloride binding. These interactions of amilorides with the amino acids of the sodium ion site are of interest because these have been shown to be important in receptor functionality, with Asp^2.50^ and Trp^6.48^ as most noticeable examples. Mutation of Asp^2.50^ silences receptor activation in many GPCRs.^84^ Trp^6.48^ is noteworthy as part of an “activation micro‐switch” between the active and inactive states of GPCRs,^31^, ^85^ and in docking studies of the adenosine A_2A_ receptor amiloride and HMA seem to toggle this amino acid from one rotamer to another. Although not very likely, amilorides may also influence the oligomerization of class A receptors. The interface for receptor dimerization often involves transmembrane domains 4 and 5 that are not part of the sodium ion binding site. In some cases, however, other domains such as TM6, which also flanks the sodium ion binding site, play a role.^86^

In contrast with these general “rules,” differences in the affinities, potencies, and modulatory behaviors of amilorides can be quite outspoken, even between receptors where the sodium ion site harbors the same amino acids (i.e. adenosine, adrenergic, dopamine, and muscarinic receptors). To appreciate these differences it is important to discern between the different properties by which the allosteric effect of amilorides on orthosteric ligand binding may be described. In Table 1 we collected values for the different amilorides, of their affinity in displacing orthosteric ligands (IC_50_ or K_i_), their (allosteric) effect on the dissociation of orthosteric ligand (k_off_/k_off(control)_), and their potency for these dissociation effects (EC_50_). This information also helps to understand whether the interaction of a particular amiloride with an orthosteric ligand is competitive or noncompetitive. If amiloride inhibits orthosteric ligand binding but does not affect its dissociation rate, the binding is mutually exclusive and the interaction is defined as competitive. If the dissociation rate is changed though, both the orthosteric ligand and amiloride can bind to the receptor at the same time and the interaction is deemed noncompetitive. Another way to confirm a noncompetitive interaction is by showing insurmountability of the inhibiting effect in radioligand saturation (B_max_ decrease) or functional assays (E_max_ decrease), as discussed for the chemokine CCR2, muscarinic M_3_, and gonadotropin‐releasing hormone receptor. However, these assays have been conducted far less than dissociation assays in amiloride research so we did not include these in Table 1.

In some cases, amilorides behave only as purely competitive inhibitors, whereas in other cases they behave as noncompetitive negative modulators, and a mixed behavior has also been observed. For some receptors the cause for mixed competitive/noncompetitive behavior was explained by a tendency of amilorides to bind both orthosteric and allosteric sites, but also in these cases the observed effect may be caused by binding in the sodium ion site only, where the competitive “fraction” of the allosteric effect is caused by either an overlap of binding with the orthosteric site or a conformational change of the receptor by amiloride binding. The latter option is quite likely from the structural evidence provided by the recently elucidated crystal structures.

At some of the discussed receptors, the modulatory effect by amilorides is probe‐dependent, which has been described in other cases of allosteric modulation as well.^87^, ^88^ Amilorides act as positive allosteric modulators for agonist binding and as negative modulators for antagonists at the α_2A_‐adrenergic and adenosine A_3_ receptors. Thus, in some cases, amilorides may also influence receptor signaling after agonist activation with consequences for effector bias or functional selectivity, for instance between G protein and β‐arrestin signaling.^89^, ^90^ This has, however, not been demonstrated yet. At the α_2B_‐adrenergic receptor different amilorides even exhibit both positive and negative modulatory effects on the same orthosteric probe. Some of the differences in affinity and modulatory effect may be caused by differences in the sodium ion site itself, but the substantial conservation of the sodium ion site residues amongst GPCRs makes it more likely that these differences are caused by variations in receptor conformations.

Clinical application of amilorides targeting GPCRs is not self‐evident due to their micromolar affinities and lack of selectivity. However, it may be feasible to synthesize amiloride analogs with variations on the 5′‐position to improve their affinity and selectivity for GPCRs. In that sense, the recent structure elucidation of the BLT1/leukotriene B_4_ receptor in complex with BILL260 (Figure 8) is noteworthy. BIIL260 is a selective, high‐affinity antagonist for this receptor, occupying both the sodium ion and the orthosteric binding site. With the ongoing expansion of the crystal structure pool of GPCRs, further study and knowledge of the mechanisms of amiloride modulation will help in understanding and appreciating the allosteric mechanism in GPCR functioning and may pave the way for the design of antagonists forcing the receptor in a deeply inactive state.
